# Pellino-1 Regulates Immune Responses to *Haemophilus influenzae* in Models of Inflammatory Lung Disease

**DOI:** 10.3389/fimmu.2019.01721

**Published:** 2019-07-31

**Authors:** Bethany M. Hughes, Charlotte S. Burton, Abigail Reese, Maisha F. Jabeen, Carl Wright, Jessica Willis, Nika Khoshaein, Elizabeth K. Marsh, Peter Peachell, Shao C. Sun, David H. Dockrell, Helen M. Marriott, Ian Sabroe, Alison M. Condliffe, Lynne R. Prince

**Affiliations:** ^1^Department of Infection, Immunity and Cardiovascular Disease, University of Sheffield, Sheffield, United Kingdom; ^2^Department of Immunology, The University of Texas MD Anderson Cancer Center, Houston, TX, United States; ^3^MRC Centre for Inflammation Research, University of Edinburgh, Edinburgh, United Kingdom

**Keywords:** *Haemophilus influenzae*, Pellino-1, immunity, lung, inflammation

## Abstract

Non-typeable *Haemophilus influenzae* (NTHi) is a frequent cause of lower respiratory tract infection in people with chronic obstructive pulmonary disease (COPD). Pellino proteins are a family of E3 ubiquitin ligases that are critical regulators of TLR signaling and inflammation. The aim of this study was to identify a role for Pellino-1 in airway defense against NTHi in the context of COPD. Pellino-1 is rapidly upregulated by LPS and NTHi in monocyte-derived macrophages (MDMs) isolated from individuals with COPD and healthy control subjects, in a TLR4 dependent manner. C57BL/6 *Peli1*^−/−^ and wild-type (WT) mice were subjected to acute (single LPS challenge) or chronic (repeated LPS and elastase challenge) airway inflammation followed by NTHi infection. Both WT and *Peli1*^−/−^ mice develop airway inflammation in acute and chronic airway inflammation models. *Peli1*^−/−^ animals recruit significantly more neutrophils to the airway following NTHi infection which is associated with an increase in the neutrophil chemokine, KC, in bronchoalveolar lavage fluid as well as enhanced clearance of NTHi from the lung. These data suggest that therapeutic inhibition of Pellino-1 may augment immune responses in the airway and enhance bacterial clearance in individuals with COPD.

## Introduction

COPD will be the third-leading cause of death worldwide by 2030 (1). Recurrent bacterial and viral infections are a significant cause of comorbidity in COPD, resulting in accelerated decline in lung function and posing a major economic and personal burden (2). The most common colonizing microorganism is NTHi, found in the lower respiratory tract of 30% of individuals with COPD (3). This, as well as the acquisition of new strains of NTHi, are important causes of acute exacerbations and NTHi directly contributes to airway inflammation in disease (4–6). The primary cellular immune response to NTHi is the alveolar macrophage (AM), but in COPD, they often fail to clear bacteria from the airway (7). NTHi infection leads to a Toll-like receptor (TLR)-dependent immune response in AMs, including the release of proinflammatory cytokines such as CXCL8 (8), which recruits neutrophils to the lung. Neutrophils are an early and prominent component of the immune response in the lung to NTHi infection, as exemplified in murine models (9) and TLR-mediated neutrophil recruitment is likely to be an integral part of the airway response to NTHi.

Pellino proteins are a family of E3 ubiquitin ligases that play important roles in TLR signaling and human immunity (10). Increased Pellino-1 expression is associated with persistent bacterial infection of the airways (11). Pellino-1 knockout mice develop normally but are protected from the adverse effects of systemic administration of TLR3 and TLR4 agonists, and Pellino-1 is shown to mediate endotoxin tolerance, further supporting its importance in infection-related inflammation (12, 13). The aim of this study was to identify a role for Pellino-1 in airway defense against NTHi in the context of COPD. Here we show that induction of Pellino-1 is part of the cellular response to NTHi *in vitro* and negatively regulates bacterial clearance *in vivo*. Our work suggests that Pellino-1 is a key component of the immune response to NTHi in the airway and that therapeutically targeting Pellino-1 may enhance immunity in patients with COPD who are at risk of infection induced exacerbations.

## Materials and Methods

### Animals

All work involving animals was performed in accordance with the Animal (Scientific procedures) Act 1986 and has been approved by the Animal welfare and ethical review body at University of Sheffield. Work was carried out under procedure project license 40/3726 (David Dockrell). C57BL/6 *Peli1*^−/−^ mice and WT littermates (13) were maintained via het/het breeding in a pathogen-free environment and were housed in shared cages. For full details of maintenance and experimental procedures see online Supplementary Methods File. COPD model: Mice were intranasally exposed to 7μg LPS (*E. coli* O26:B6, Sigma-Aldrich (St. Louis, MO) and 1.2 units elastase (Merck Millipore, Burlington, MA) each week for four consecutive weeks as previously described (14). Selected mice were treated with PBS as controls. All mice were randomized into treatment groups. On day 28 mice were subjected to bronchoalveolar lavage (BAL). Cell-free BAL fluid (BALF) was prepared and cell pellets were resuspended, counted using a haemocytometer and cytocentrifuge slides generated. Neutrophils and macrophages were identified based on cell morphology. COPD NTHi infection model: Mice were treated with weekly LPS/elastase as described above. On day 28 mice were infected with NTHi375 (15) (10^7^ CFU i.n.) for 24 h. Following this, mice were subjected to BAL as above and lungs homogenized to measure bacterial viability by Miles and Misra viability counts. Acute lung injury LPS model: *Peli1*^−/−^ and WT mice were exposed to 7μg LPS i.n. After 24 h mice were infected with NTHi375 (10^7^ CFU i.n.) for a further 24 h. Mice were subjected to BAL, lung homogenisation and estimation of NTHi CFU counts as above. Bone marrow derived macrophages (BMDMs) were prepared as previously described (16). Bone marrow derived neutrophils (BMDNs) were isolated by negative magnetic selection (EasySep Mouse neutrophil enrichment kit, Stemcell Technologies) as per manufacturer's recommendation. Following negative selection neutrophil purity was typically >95%.

### Human Subjects

Peripheral blood was taken from healthy volunteers, people with a diagnosis of COPD, or age-matched healthy control (AMHC) subjects with written informed consent as per the declaration of Helsinki, and ethical approval in accordance with the recommendations of the South Sheffield Research Ethics Committee and the National Research Ethics Service Committee Yorkshire and the Humber (17). See Table 1 for demographics of individuals with COPD and AMHC subjects.

**Table 1:** Demographics of individuals with COPD and AMHC subjects.

**Demographic**	**COPD (*n* = 3)**	**AMHC (*n* = 3)**
Age (mean years)	61	63
Gender (m/f)	3/0	2/1
FEV_1_ % mean (range)	50.7 (30–71)	103 (87–113)
GOLD stage	2-3	NA
ICS	No (3)	No (3)
Smoking status	Current (1), ex (2)	Never (3)

*FEV_1_, forced expiratory volume in 1 second; ICS, inhaled corticosteroids*.

### Monocyte-Derived Macrophage (MDM) Isolation and Culture

Neutrophils and mononuclear cells were isolated by plasma-Percoll gradient centrifugation from whole blood. MDMs were differentiated over 7 days as previously described (18). *Peli1* was knocked down in MDMs using Dharmacon ON-TARGET plus SMARTpool^TM^ siRNA and Lipofectamine 2000 (ThermoFisher Scientific), according to manufacturer's instructions. Knockdown of Pellino-1 protein was verified in each experiment by Western Blotting.

### Isolation and Culture of AMs From Human Lung

The use of lung tissue was approved by the National Research Ethics' Service (REC ref:15/NW/0657) and informed written consent was obtained. Macrophages were isolated from resected non-lesional tissue by discontinuous Percoll gradient sedimentation as previously described (19). Macrophages were seeded and cultured overnight in media (RPMI 1640+10% FCS) before LPS challenge.

### Western Blotting

Proteins in whole cell lysates were separated by SDS-polyacrylamide gel electrophoresis and transferred onto PVDF membranes. Membranes were probed against antibodies to Pellino-1, actin (Santa Cruz, Santa Cruz, CA), pStat-1 or Stat-6 (Cell-Signaling Technology, Leiden, The Netherlands).

### Measurement of KC

Cell free BALF and cell culture supernatant were subjected to KC ELISA (Duoset, R&D Systems) as per manufacturer's instructions. Formalin fixed lung sections from LPS/elastase treated mice were subjected to immunohistochemistry for KC.

### NTHi Intracellular Viability Assays

Cells were infected with NTHi375 (MOI 10) for either 1 h (BMDNs) or 2 h (MDMs, BMDMs). Cells were lysed and viable NTHi measured by Miles and Misra assay. In separate wells, gentamicin [40 μg/ml] was added for 30 min to kill extracellular bacteria following which lysates were made after a further 1 h (neutrophils) or 2 h (MDMs, BMDMs) and viable intracellular NTHi was assessed as above.

### Data Analysis and Statistics

Data were analyzed by One-way ANOVA (with post-test) or Students' *t*-test as appropriate using GraphPad Prism 7 (GraphPad Software, San Diego, CA). Data are expressed as mean ± SEM or mean ± SD, and significance was accepted at *p* < 0.05.

## Results

### Macrophages Upregulate Pellino-1 in Response to LPS and NTHi via TLR4

**Figure 1:**
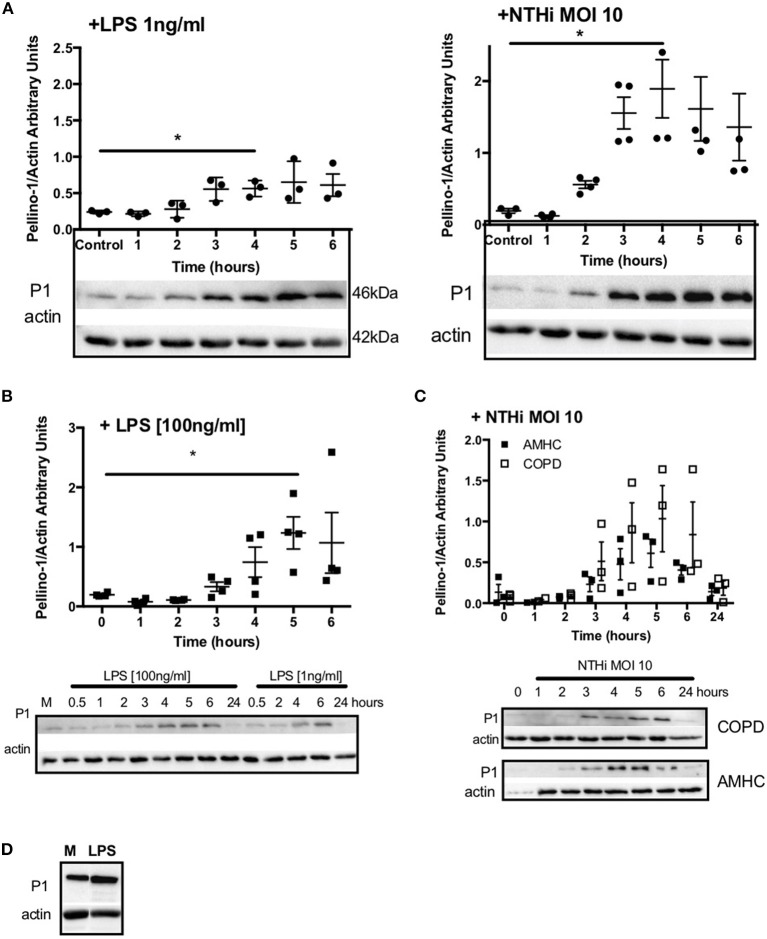
Macrophages upregulate Pellino-1 in response to LPS and NTHi. MDMs were stimulated with LPS [1ng/ml] or NTHi (MOI 10) **(A)** for 1-6 h. Cells were also cultured in media for 6 h (control, **A**). MDMs differentiated from monocytes isolated from a COPD patient were treated with LPS at 1 or 100 ng/ml **(B)**, or NTHi MOI 10 **(C)** over a time course of 24 h. MDMs from age-matched healthy control subjects (AMHC) were challenged with NTHi in matched experiments **(C)**. Primary human neutrophils were treated with LPS [1ng/ml] for 6 h **(D)**. Lysates for all cells were prepared and subjected to Western blotting using antibodies to Pellino-1 (P1) or actin (loading control). Densitometry was performed on independent donors/experiments and data are presented as mean ± SEM. Panel d is representative of 3 experiments. Statistically significant differences are indicated by ^*^p ≤ 0.05.

**Figure 2:**
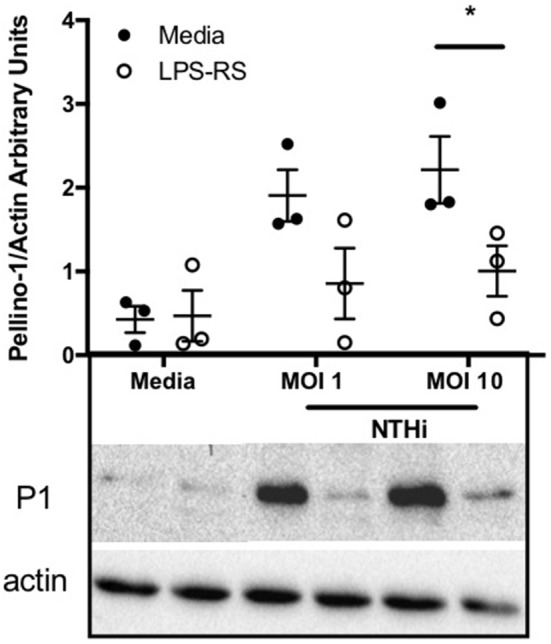
NTHi-mediated upregulation of Pellino-1 occurs via TLR4. MDMs were stimulated with NTHi (MOI 1 or 10), or media control for 6 h in the presence (open bars) or absence (black bars) of the TLR4 antagonist LPS-RS [1 μg/ml]. Lysates were prepared and subjected to Western blotting using antibodies to Pellino-1 (P1) or actin (loading control). Representative blot as shown in inset. Densitometry was performed on 3 independent donors/experiments and data are presented as mean ± SEM. Statistically significant differences are indicated by ^*^p ≤ 0.05.

Since Pellino-1 has known roles in TLR4 signaling in monocytes (12), we explored Pellino-1 regulation in response to LPS and NTHi. In MDMs from healthy subjects, Pellino-1 protein is profoundly upregulated by LPS and NTHi (Figure 1A). This was confirmed in MDMs prepared from people with COPD and age-matched healthy control subjects (Figures 1B,C). Since macrophage phenotype can be sensitive to *in vitro* differentiation programmes, we also confirmed an LPS-dependent upregulation of Pellino-1 in preliminary work in primary AMs isolated from human lung (Supplemental Figure 1A). Primary human neutrophils also express Pellino-1 (Figure 1D). In contrast, Pellino-1 protein is not regulated by the Gram-positive bacteria, *Staphylococcus aureus* and *Streptococcus pneumoniae*, nor the TIR agonist IL-1β (Supplemental Figures 1B–D) although MDMs released CXCL8 in response to these stimuli (data not shown). Although LPS signals exclusively via TLR4, NTHi can also activate TLR2 in macrophages. Figure 2 shows that the TLR4 antagonist LPS-RS (20) significantly reduces NTHi-induced upregulation of Pellino-1, suggesting NTHi signals, at least in part, via TLR4 to induce Pellino-1 expression.

### *Peli1^−/−^* Mice Develop Airway Inflammation in Models of COPD

**Figure 3:**
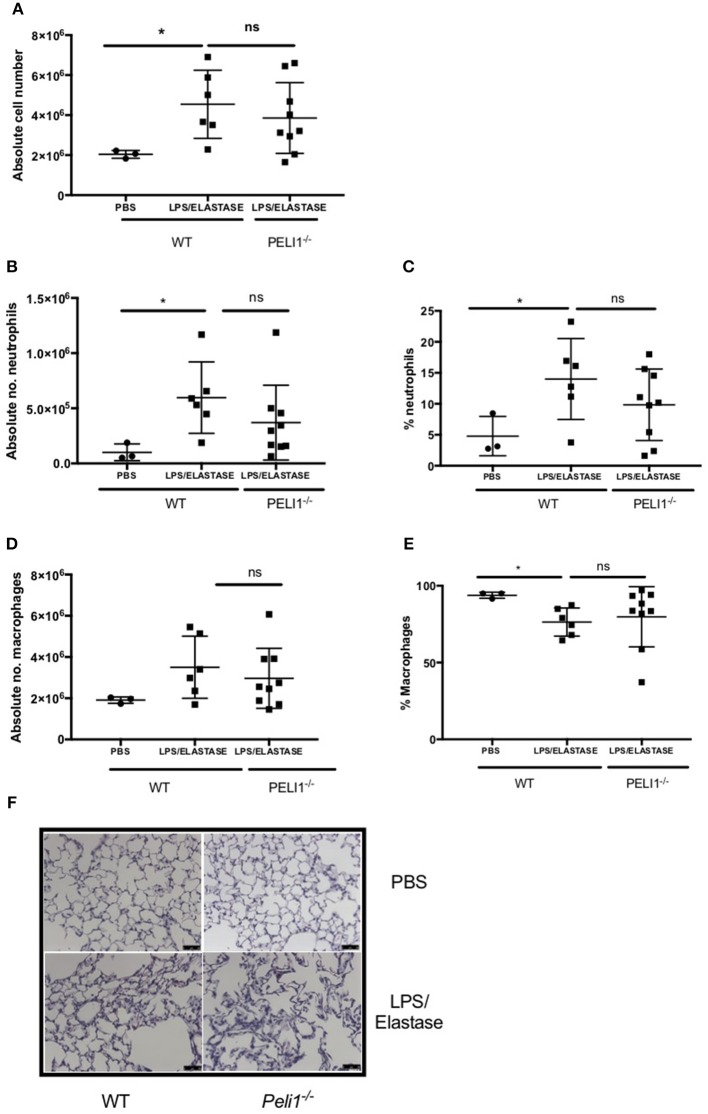
WT and *Peli1*^−/−^ mice develop airway inflammation in response to repeated LPS and elastase. *Peli1*^−/−^ transgenic mice and WT littermates were given i.n. PBS (black circles) or LPS (7μg) and porcine neutrophil elastase (1.2 units, black squares) every 7 days for 4 weeks. Mice were subjected to bronchoalveolar lavage (BAL) on day 28 and inflammation assessed by enumerating total cell number in BALF by haemocytometer **(A)** and number/proportion of neutrophils **(B,C)** and macrophages **(D,E)** by light microscopy. Lungs were removed and stained for histological analysis. Representative images are shown (**F**, scale bar = 50 μm). Individual data points represent a single mouse and panels show mean ± SD. Statistically significant differences are indicated by ^*^p < 0.05 (*n* = 3–5 **A**, *n* = 3–9 **B–E**).

To establish a role for Pellino-1 in lung inflammation and infection, we adopted a murine model of COPD in Pellino-1 (*Peli1*^−/−^) knockout mice and WT littermates (13, 14). *Peli1*^−/−^ and WT mice were treated with LPS and elastase (or PBS as control) once per week for 4 weeks. As expected, there was an increase in inflammatory cells in the WT LPS/elastase BALF compared to WT PBS treated mice (Figure 3A). The proportion and absolute number of neutrophils (Figures 3B,C) and macrophages (Figures 3D,E) in BALF was increased in WT LPS/elastase mice compared to WT PBS animals. No significant difference was observed between WT and *Peli1*^−/−^ LPS/elastase treated animals for either absolute number or proportion of cells by type (Figures 3A–E). WT and *Peli1*^−/−^ mice treated with PBS were comparable for all cell counts (data not shown). Lung histology from PBS-treated WT and *Peli1*^−/−^ mice shows no evidence of inflammation (Figure 3F, upper panels). Both WT and *Peli1*^−/−^ LPS/elastase-treated mice developed airway inflammation, illustrated by an increase in cellularity and loss of alveolar architecture consistent with emphysema (Figure 3F, lower panels). These findings show that loss of Pellino-1 does not impact on the development of airway inflammation in this model at the timepoint studied.

### *Peli1^−/−^* Mice Recruit More Neutrophils to the Airway in Response to NTHi and Clear the Infection More Effectively in Murine Models of COPD

**Figure 4:**
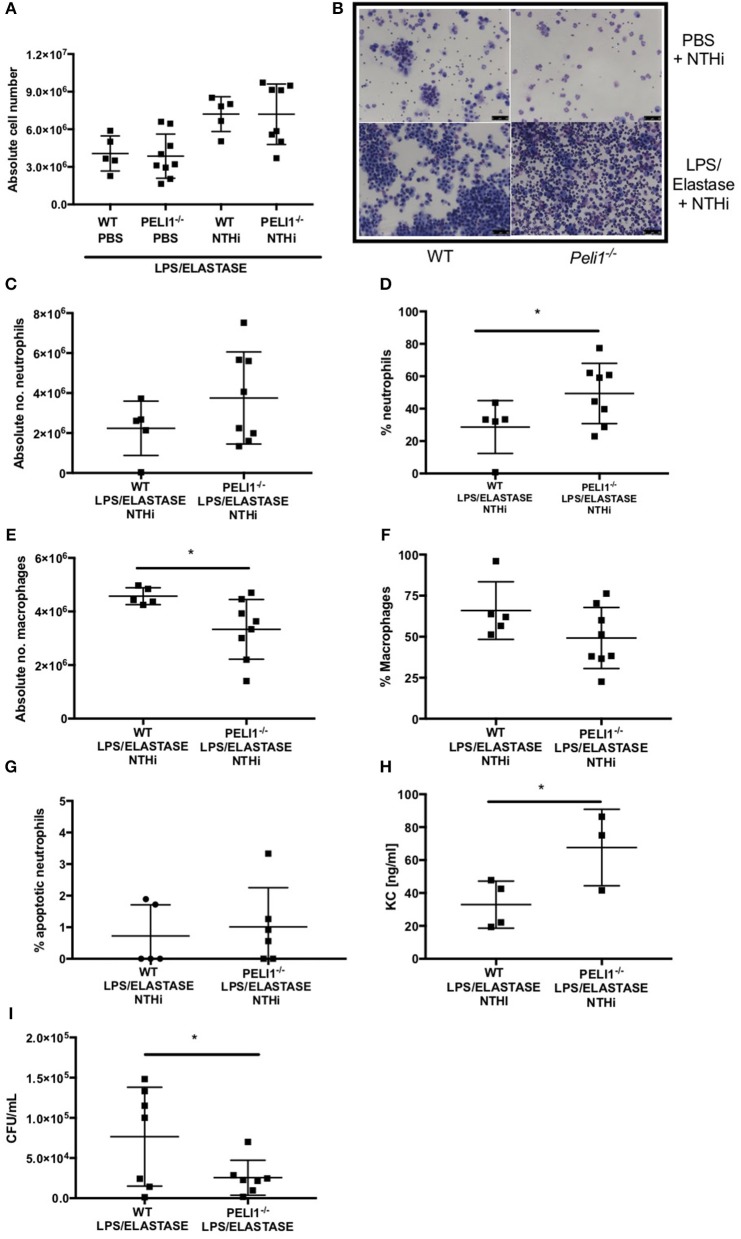
*Peli1*^−/−^ COPD mice recruit more neutrophils following NTHi infection and clear NTHi more effectively compared to WT. *Peli1*^−/−^ transgenic mice and WT littermates were given i.n. LPS [7μg] and 1.2 units porcine neutrophil elastase every 7 days for 4 weeks. On day 28, mice were infected with 1 × 10^7^ CFU NTHi. After 24 h (day 29) lungs were lavaged and removed for homogenisation. Total cell number in BALF was enumerated by haemocytometer **(A)** and cell differential determined by light microscopy of cytocentrifuge slides, scale bar = 50 μm **(B)**, Number/proportion of neutrophils **(C,D)** and macrophages **(E,F)** were determined. Percentage of apoptotic neutrophils were scored on BALF cytocentrifuges by morphology **(G)**. KC levels in BALF were measured by ELISA **(H)** and NTHi CFU in homogenized lungs was determined by Miles Misra **(I)**. Individual data points represent a single mouse and panels show mean ± SD. Statistically significant differences are indicated by ^*^p < 0.05 (*n* = 4–8 **A**, *n* = 5–8 **C–F**, *n* = 5-6 **G** (apoptosis), *n* = 3–4 h (KC), *n* = 7 **I** (NTHi)).

WT and *Peli1*^−/−^ mice pre-treated with LPS/elastase were infected with NTHi (or PBS as control. Both WT and *Peli1*^−/−^ mice mounted an immune response to NTHi, as demonstrated by an increase in absolute cell number in BALF (Figure 4A). There was no significant difference in absolute cell number between WT and *Peli1*^−/−^ NTHi infected animals (Figure 4A). NTHi infected *Peli1*^−/−^ mice have proportionally more neutrophils in BALF compared with WT (Figure 4B, lower panels and Figures 4C,D). This was concomitant with a decrease in the number of macrophages (Figures 4E,F). Neutrophil apoptosis was assessed on BALF cytocentrifuge slides and there was no difference between WT and *Peli1*^−/−^ mice (Figure 4G). The principal neutrophil chemokine, KC, was significantly increased in BALF from *Peli1*^−/−^ mice (Figure 4H). CFU counts from homogenized lungs show fewer viable bacteria from *Peli1*^−/−^ mice compared to WT mice (Figure 4I). No viable bacteria were grown from blood cultures (data not shown).

### Increased Airway Neutrophilia in *Peli1^−/−^* Mice Following Acute Lung Injury and NTHi Infection

**Figure 5:**
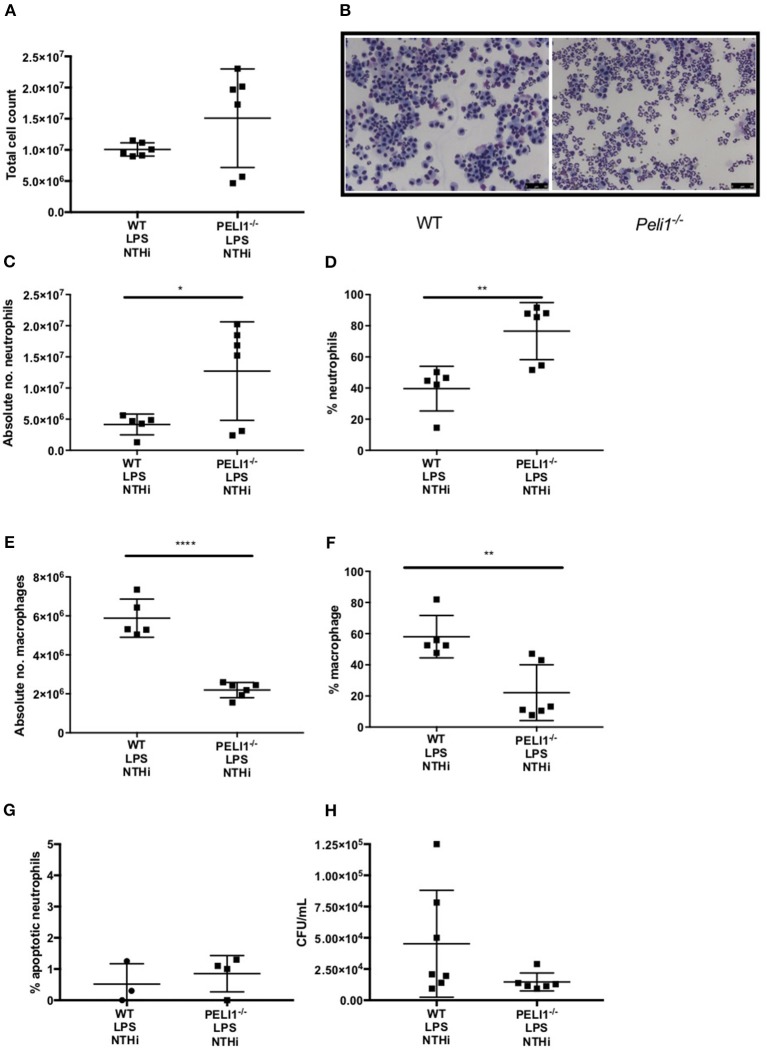
*Peli1*^−/−^ acute lung injury mice recruit more neutrophils following NTHi infection and clear NTHi more effectively compared to WT. *Peli1*^−/−^ transgenic mice and WT littermates were given a single treatment of i.n. LPS [7μg] (day 1). After 24h mice were infected with 1x10^7^ CFU NTHi (day 2). After 24h (day 3) lungs were lavaged and subsequently removed for homogenisation. Total cell number in BALF was enumerated by haemocytometer **(A)** and cell differential determined by light microscopy of cytocentrifuge slides, scale bar = 50 μm **(B)**. The number/proportion of neutrophils **(C,D)** and macrophages **(E,F)** were determined. Percentage of apoptotic neutrophils were scored on BALF cytocentrifuges by morphology **(G)**. NTHi CFU in homogenized lungs was determined by Miles and Misra method **(H)**. Individual data points represent a single mouse and panels show mean ±SEM. Statistically significant differences are indicated by ^*^p < 0.05, ^**^p < 0.01, ^****^p < 0.0001 *n* = 6 **A**, *n* = 5–6 **C–F**, *n* = 3–4 **G** (apoptosis) *n* = 6-7 **D** (NTHi).

These findings were recapitulated in an LPS acute lung injury model, which typically results in a more marked neutrophilic inflammation (21). As before, the overall cell number in BALF was not different between WT and *Peli1*^−/−^ mice (Figures 5A,B). Both the number and proportion of neutrophils were significantly increased in *Peli1*^−/−^ mice (Figures 5C,D) and concomitantly, the number and proportion of macrophages were significantly decreased (Figures 5E,F). There was no difference in the rate of neutrophil apoptosis in BALF between WT and *Peli1*^−/−^ mice (Figure 5G). A trend toward fewer viable NTHi was observed in lungs of *Peli1*^−/−^ mice compared to WT mice, although this did not reach statistical significance, potentially due to sample size (*P* = 0.056, Figure 5H). Overall, these data suggest that suppression of Pellino-1 leads to improved bacterial clearance.

### Loss of Pellino-1 Does Not Impact on Killing or Internalization of NTHi by Neutrophils and Macrophages

**Figure 6:**
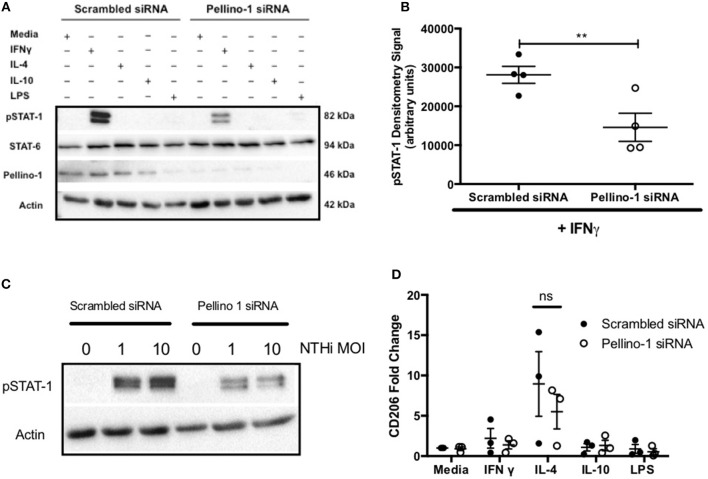
Pellino-1 regulates macrophage STAT-1 signaling. MDMs were transfected with siRNA targeting Pellino-1 or scrambled siRNA (control). **(A,B,D)** 48 h following transfection, cells were stimulated with IFN-γ [20 ng/ml], IL-4 [20 ng/ml], IL-10 [20 ng/ml] or LPS [1 ng/ml] for 24 h. Whole cell lysates were analyzed by Western blot using antibodies specific to STAT-1, pSTAT-6, Pellino-1 or actin for 4 independent donors **(A)**. pSTAT-1 bands for IFN-γ-treated cells were analyzed by densitometry **(B)**. In separate experiments cells were challenged with NTHi (MOI 1 and 10) for 24 h and lysates probed for Pellino-1 and actin **(C)**. RNA was purified from cell lysates and transcribed to cDNA. *CD206* gene product was measured by qPCR **(D)**. Fold changes are shown relative to media treated MDMs transfected with scrambled siRNA (first open bar). Data are presented as mean ± SEM of *n* = 4 independent experiments. Significant differences are indicated by ^**^P < 0.01.

Based on known roles for Pellino-1 in macrophage polarization we next studied whether the enhanced clearance of NTHi in *Peli1*^−/−^ mice was due to promotion of a particular macrophage phenotype (22). To do this we used an *in vitro* siRNA approach in MDMs. STAT-1 and STAT-6 transcription factors facilitate distinct macrophage phenotypes and are activated by LPS/IFNγ and IL-4/IL-10, respectively (22, 23). IFNγ, but surprisingly not LPS, upregulated phosphorylated STAT-1 (pSTAT-1) (Figure 6A), which was significantly reduced in cells transfected with *Peli1* siRNA (Figure 6B). STAT1 is phosphorylated following TLR2 and TLR4 activation in macrophages (24) and here we show that NTHi induces pSTAT-1, which is reduced in *Peli1* siRNA transfected cells (Figure 6C). IL-4 failed to upregulate STAT-6 (Figure 6A) therefore we measured CD206 expression, which is regulated by STAT-6, and show it is upregulated by IL-4, but unaffected by *Peli1* knockdown (Figure 6D).

**Figure 7:**
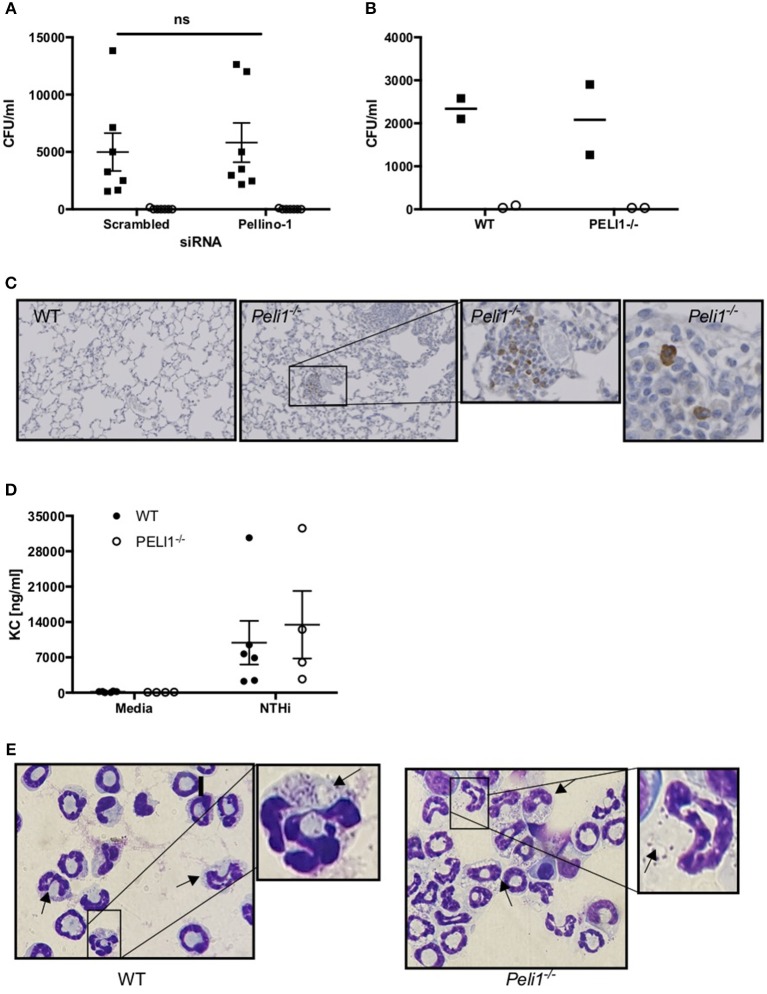
Internalization and killing of NTHi is not impaired by loss of Pellino-1. **(A)** MDMs were transfected with siRNA targeting Pellino-1 or scrambled siRNA (control). 48 h following transfection, MDMs were infected with NTHi (MOI 10) for 2 h after which cells were lysed and viable intracellular bacteria enumerated by Miles Misra (2 h, black squares). In parallel wells, MDMs were treated with gentamycin to kill extracellular bacteria for a further 2 h, after which intracellular NTHi were enumerated by Miles Misra (4 h, open circles). **(B)** WT and *Peli1*^−/−^ BMDM were infected with NTHi (MOI 5) and viable bacteria enumerated at 2 and 4 h as above. **(C)** Lung sections from WT and *Peli1*^−/−^ mice treated with LPS/Elastase were subjected to histology and immunostained for KC. Zoomed inset shows individual cells staining for KC in brown. **(D)** Day 14 WT and *Peli1*^−/−^ BMDM were infected with NTHi (MOI 5) for 24 h and cell free supernatants subjected to KC ELISA. **(E)** neutrophils were isolated from WT and *Peli1*^−/−^ BMDM and infected with NTHi (MOI 10) for 1h. Cytocentrifuge slides were prepared and arrows point to neutrophils containing NTHi. Zoomed inset highlights NTHi within intracellular vacuoles. Data are expressed as individual points with mean±SEM (**A**, *n* = 7) or mean (**B**, *n* = 2) or as bars (**C**, mean±SEM, *n* = 3–5).

These data suggest that loss of Pellino-1 does not promote an anti-microbial macrophage phenotype. This was confirmed in NTHi killing assays where both scrambled and *Peli1* siRNA transfected MDMs were able to internalize (assayed at 2 h) and entirely eradicate (assayed at 4 h) NTHi to a comparable degree (Figure 7A). This was supported in BMDMs prepared from WT and *Peli1*^−/−^ mice (Figure 7B). Murine macrophages are a source of KC and synthesis of this chemokine occurs via TLR-mediated pathways. Histology on lung sections from LPS/elastase treated WT and *Peli1*^−/−^ mice show individual cells staining for KC (Figure 7C). Since these cells were of a macrophage like appearance and since we measured increased KC in BALF from *Peli1*^−/−^ mice (Figure 4D), we investigated KC production from BMDMs from WT and *Peli1*^−/−^ mice. Figure 7D shows no difference in NTHi-induced KC production by *Peli1*^−/−^ BMDMs compared to WT.

Since *Peli1*^−/−^ macrophages were no more effective at killing NTHi *in vitro*, we hypothesized that it is the increased neutrophil number in the lung of *Peli1*^−/−^ mice that results in enhanced bacterial clearance. Neutrophils can phagocytose and destroy NTHi (8) and we examined whether Pellino-1 knockout impacted on this neutrophil function. No viable bacteria were recovered from either WT nor *Peli1*^−/−^ bone marrow-derived neutrophils at any timepoint studied, despite being able to visualize NTHi inside these cells (Figure 7E). These data demonstrate that despite loss of Pellino-1, neutrophils rapidly and completely kill internalized NTHi, and suggest it is feasible that increased neutrophil number in *Peli1*^−/−^ mice leads to better clearance of NTHi.

## Discussion

We show that human macrophages rapidly and profoundly upregulate Pellino-1 in response to LPS and NTHi, suggesting a potential role for this protein in the initial cellular immune response to NTHi. In *in vivo* models of acute and chronic inflammation, *Peli1*^−/−^ mice recruit more neutrophils to the airway following NTHi challenge, compared to WT animals. This is accompanied by an increase in the neutrophil chemoattractant KC in BALF. *Peli1*^−/−^ mice clear NTHi more effectively from the lung than WT littermates, which may be as a result of the increased neutrophilic immune response in the airway. Our work suggests that Pellino-1 is a key component of the airway immune response to NTHi and that therapeutically targeting Pellino-1 may enhance clearance of NTHi in patients with chronic inflammatory disease who are at risk of infection induced exacerbations.

Pellino-1 interacts with TIR signaling pathways at various points due to its ability to both mark proteins for degradation (via K48-linked ubiquitination) and initiate downstream signaling (via K63-linked ubiquitination) (25, 26). It has both negative and positive regulatory roles on signaling, depending on the cell type and stimulus (27, 28). Our previous work identifies a role for Pellino-1 in epithelial cell responses to rhinoviral infection (29) and this along with a growing literature on roles for Pellino-1 in infection (30), led us to hypothesize that loss of Pellino-1 would impact on responses to the airway pathogen, NTHi. The initial cellular immune response to NTHi is predominantly mediated by AMs, followed by recruitment of neutrophils, which primarily function during acute infective exacerbations (8, 31). Studies have shown that macrophages from people with COPD are defective in phagocytosing NTHi (32). Since healthy lungs are mostly resistant to NTHi infection and murine models also show rapid clearance, we adopted a murine model of COPD, which better mimics host-pathogen interactions in patients with this disease (33). LPS/elastase-induced lung inflammation occurred irrespective of genotype. This suggested that although Pellino-1 is a component of the TLR4 pathway it was not essential for mediating inflammatory signals via LPS in this context, perhaps indicating some functional redundancy in this pathway. NTHi infection resulted in additional neutrophil recruitment to the lung, confirming findings from similar studies (33), and which is observed in people with COPD colonized with NTHi (34). *Peli1*^−/−^ mice had more neutrophils and fewer macrophages in BALF, which may in part be accounted for by an increase in KC. The source of the KC is not clear, and while resident tissue macrophages are thought to be a principal source of KC in the murine lung, epithelial cells also produce this chemokine in concert with airway myeloid cells following infection (35). Lung histology images suggest cells with a macrophage-like morphology rather than epithelial cells appear to be more intensely stained for KC. *Peli1*^−/−^ BMDMs show a trend for increased KC production in response to NTHi *in vitro*, although this is not statistically significant, but may indicate a negative regulatory role for Pellino-1. Although Pellino-1 has previously been identified as a positive regulator of macrophage TLR signaling (27), it negatively regulates T cell activation (28). Th17 cells play an important role in neutrophil recruitment to the airway following NTHi infection (31), and heightened T cell activation in *Peli1*^−/−^ mice may be the cause of the airway neutrophilia we see in our model. Pellino also negatively regulates immunity in *Drosophila* and ablation of Pellino in adult flies promotes clearance of *Micrococus luteus* (36). A similar role has been described for Pellino-3, where knockdown results in increased inflammatory cytokine production following bacterial infection of macrophages (12). Evidence for Pellino-1 in the negative regulation of inflammation is found in a murine ischemic skin flap study, where Pellino-1 overexpression leads to reduced infiltration of inflammatory lymphocytes to the dermis and faster resolution of inflammation (37). Moreover, the Smad-6 interacting peptide, Smaducin-6, disrupts Pellino-1 signaling and has been shown in murine models of sepsis to improve bacterial clearance via increased neutrophil recruitment, supporting a role for Pellino-1 in negatively regulating immunity in the context of bacterial infections (38). Our findings may suggest a way to improve immunity in patients with COPD, which may be made possible by development of tools such as Smaducin-6. These observations are also interesting in that reducing neutrophilic inflammation because of its lung damaging potential in COPD is often considered to be a plausible therapeutic target: our data indicate that in people with chronic infection, reduction in lung neutrophil numbers may not always be a desirable aim. Whether inducing an airway neutrophilia is detrimental for patients with inflammatory disease in this way would need further study.

In our models, it is most likely that increased bacterial clearance is consequent upon neutrophil recruitment, since we did not show increased monocyte recruitment. Furthermore, although we showed some decrease in polarization markers in Pellino-1 knockout macrophages, macrophage killing of NTHi was unimpaired. Others have shown that Pellino1 promotes macrophage polarization *in vitro*, which is in support of the STAT-1 downregulation we observed in Pellino1 siRNA transfected MDMs (39). We would therefore expect loss of Pellino1 to result in impaired killing of NTHi, but we could not find a difference in the internalization or killing capacity of Pellino1 deficient human or murine macrophages, which may indicate eradication of NTHi occurs irrespective of polarization state.

We show Pellino-1 expression is linked to TLR4 signaling and our data support previous studies showing Pellino-1 is regulated in response to LPS (12). TLR4 knockout mice have reduced inflammation and impaired bacterial clearance during NTHi infection, further supporting a role for neutrophils in the eradication NTHi (40, 41). In *Drosophila*, ablation of Pellino in adult flies promotes clearance of bacteria (36), in comparison with Toll mutant flies who are profoundly vulnerable to infection (42). Taken together, these studies and our work indicate that Pellino-1 knockdown in a whole organism is associated with increased bacterial clearance, preserved antimicrobial signaling, and better induction of effective innate immunity. In conclusion, we demonstrate a role for Pellino-1 in mediating immune responses in the airway and suggest that therapeutic inhibition of Pellino-1 may enhance bacterial clearance in people with COPD.

## Data Availability

All datasets generated for this study are included in the manuscript and/or the Supplementary Files.

## Ethics Statement

All work involving animals was performed in accordance with the Animal (Scientific procedures) Act 1986 and has been approved by the Animal welfare and ethical review body at University of Sheffield. Work was carried out under procedure project license 40/3726 (DD). C57BL/6 Peli1^−^/^−^ mice and WT littermates (12) were maintained via het/het breeding in a pathogen-free environment and were housed in shared cages. Peripheral blood was taken from healthy volunteers, people with a diagnosis of COPD, or age-matched healthy control (AMHC) subjects with written informed consent as per the declaration of Helsinki, and ethical approval in accordance with the recommendations of the South Sheffield Research Ethics Committee and the National Research Ethics Service Committee Yorkshire and the Humber (16).

## Author Contributions

LP, IS, DD, and AC wrote the manuscript. All authors reviewed and edited drafts of the manuscript. LP, BH, CB, AR, MJ, CW, JW, NK, and EM performed the experiments. BH, CB, AR, MJ, NK, EM, HM, LP, DD, IS, and AC contributed to experimental design and data analysis. PP provided human lung tissue samples, SS provided mice, and both contributed intellectual input to the study.

### Conflict of Interest Statement

The authors declare that the research was conducted in the absence of any commercial or financial relationships that could be construed as a potential conflict of interest.

## Supplementary Material

Supplemental Figure 1 Pellino-1 is not regulated by *S. aureus*, *S. pneumoniae* or IL-1β. AMs isolated from a single lung tissue resection were treated with LPS [0.01–10 ng/ml] for 24h **(A)**. MDMs were stimulated with media, *S. aureus* **(B)**, *S. pneumoniae* **(C)** or Il-1β **(D)** for 6 h. Lysates were prepared and subjected to Western blotting using antibodies to Pellino-1 (P1) or actin (loading control). Densitometry was performed on 3 independent donors/experiments and data are presented as mean ± SEM. No statistically significant differences were found.
