# Supplementary material for: Pellino-1 Regulates Immune Responses to Haemophilus influenzae in Models of Inflammatory Lung Disease
Source: Front Immunol. 2019 Jul 31;10:1721. doi: 10.3389/fimmu.2019.01721 (PMC6685348; doi:10.3389/fimmu.2019.01721)
Supplement: Supplementary file 1 [file Table_1.DOCX]

**Supplementary Methods.**

All work involving animals was performed in accordance with the Animal (Scientific procedures) Act 1986 and has been approved by the Animal welfare and ethical review body at University of Sheffield. Work was carried out under procedure project license 40/3726 (David Dockrell). All animals were checked prior to the start of experiments by competent personal licensees (PIL), granted under Animal (Scientific procedures) Act 1986, all animals were deemed to be fit and well before the start of experiments. The breeding colony used to supply these experiments is maintained as a het x het closed colony. Each animal constitutes an experimental unit and data were blindly reviewed in all cases.

C57BL/6 Peli1^-/-^ mice and WT littermates [12] were maintained in a specific pathogen free environment in open top cages (Pre-infection) and Individually ventilated cages set to negative pressure (post-infection). All cages were provided with ecopure flakes 6 sawdust (Datesand, UK) as bedding material and paper wool (Datesand, UK) as nest building material. All cages were given environmental enrichment in the form of a red plastic mouse house (NKP plastics, UK) and were provided with forage mix (LBS biotech, UK) once a week after cage cleaning to encourage natural foraging behaviours and to reduce the stress of cage cleaning. Animals were kept in stable social groups where possible which consisted of at least 1 cage mate, rarely mice would be housed individually due to fighting. There were no more than 5 animals per cage. All animals were fed ad-lib with irradiated Teklad global 18% protein rodent diet (Envigo, UK). Mice were provided with tap water filtered to 0.1 microns’ ad-lib. Water was changed 3 times per week. All animals are kept on a 12-hour light/dark cycle. Mice were checked after treatments and infections at least every 30 minutes for a minimum of 6 hours by experienced PIL holders. All regulated procedures were performed in licensed procedure rooms under the University of Sheffield establishment licence 50/2509 granted under Animal (Scientific procedures) Act 1986. All procedures were performed after the 12-hour dark cycle (~8am). All animals used in procedures were adult between 51 and 196 days old with a mean age of 127 days and sex ratio of 4:9 (female:male). Group sizes were determined by power calculations and carried out in 3-4 independent groups.

***COPD model:*** Selected mice were randomised using a random number generator and assigned to either a treated or control group. A total of 4 independent experiments were performed within which animals were age matched. Animals in the treated group were anesthetised using gaseous inhalation of 4-5% Isoflurane (Henry Schein) mixed with oxygen (2L/min flow) and then were intranasally instilled with 7µg LPS (*E. coli* O26:B6, Sigma-Aldrich (St Louis, MO) and 1.2 units porcine elastase (Merck Millipore, Burlington, MA) in 50ul of PBS, each week for four consecutive weeks as previously described [13]. Animals assigned to the control group were anesthetised using gaseous inhalation of 4-5% Isoflurane mixed with oxygen (2L/min flow) and then were given 50ul of PBS intranasally, each week for four consecutive weeks as previously described [13]. On day 28 mice were culled by overdose of anaesthetic (pentobarbital 20% (Henry Schein, UK) 100ul volume, death was confirmed by exsanguination via the femoral artery. After confirmation of death mice were then subjected to bronchoalveolar lavage (BAL). Cell-free BAL fluid (BALF) was prepared and cell pellets were resuspended, counted using a haemocytometer and cytocentrifuge slides generated*.*

***COPD NTHi infection model:*** Mice were treated with weekly LPS/elastase as per COPD model treated group previously described. On day 28 mice were anaesthetised using gaseous inhalation of 4-5% isoflurane and infected via intranasal route with NTHi375 [14] (10^7^ CFU). On day 29 mice were euthanized using an overdose of anaesthetic, pentobarbital 20%, death was confirmed by exsanguination via the femoral artery. After confirmation of death mice were subjected to BAL as above and lungs homogenised to measure bacterial viability by Miles and Misra viability counts.

Some mice became lethargic following treatment with LPS and elastase, LPS or NTHi all mice were given heat support immediately after treatment to negate adverse effects of lowering body temperature and lethargy.

***Acute lung injury LPS model****:* Animals were randomised using a random number generator and assigned to groups. Peli1^-/-^ and WT littermate mice were anaesthetised under gaseous inhalation of 4-5% isoflurane and treated with 7ug LPS in 50ul PBS via the intranasal route. After 24 hours mice were again anesthetised as previously mentioned and infected with NTHi375 (10^7^ CFU) via the intranasal route. A further 24 hours post infection mice were euthanized as mentioned above and subjected to BAL, lung homogenisation and estimation of NTHi CFU counts as above. Bone marrow derived macrophages (BMDMs) were prepared as previously described [15]. Bone marrow derived neutrophils (BMDNs) were isolated by negative magnetic selection (EasySep Mouse neutrophil enrichment kit, Stemcell Technologies) as per manufacturer’s recommendation. Following negative selection neutrophil purity was typically >95%.

.
