# Supplementary figures and images for: Pellino-1 Regulates Immune Responses to Haemophilus influenzae in Models of Inflammatory Lung Disease
Source: Front Immunol. 2019 Jul 31;10:1721. doi: 10.3389/fimmu.2019.01721 (PMC6685348; doi:10.3389/fimmu.2019.01721)

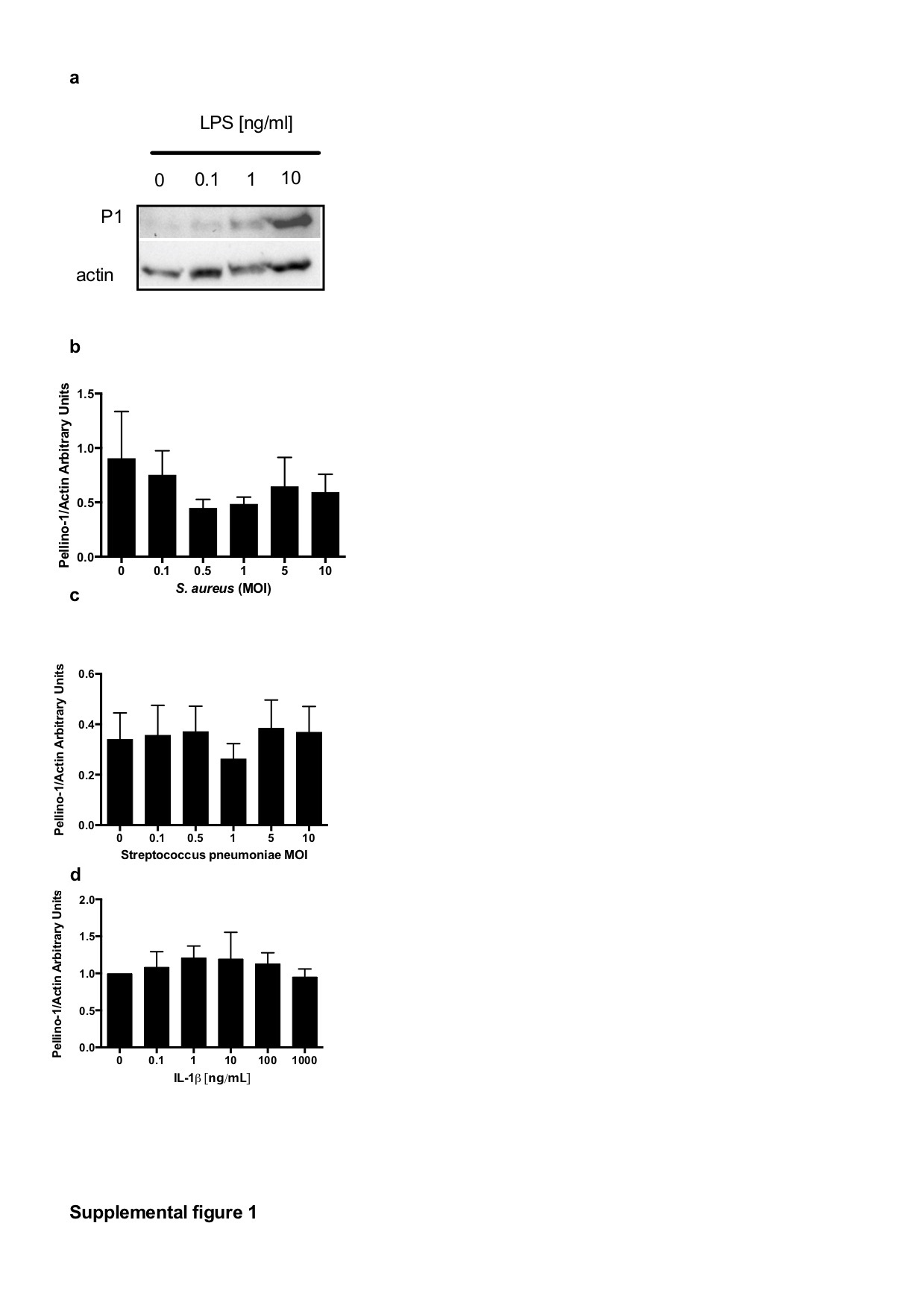

Supplement: Supplemental Figure 1 — Pellino-1 is not regulated by S. aureus, S. pneumoniae or IL-1β. AMs isolated from a single lung tissue resection were treated with LPS [0.01–10 ng/ml] for 24h (A). MDMs were stimulated with media, S. aureus (B), S. pneumoniae (C) or Il-1β (D) for 6 h. Lysates were prepared and subjected to Western blotting using antibodies to Pellino-1 (P1) or actin (loading control). Densitometry was performed on 3 independent donors/experiments and data are presented as mean ± SEM. No statistically significant differences were found. [file Image_1.jpg]
